# Severe early-onset manifestations of generalized arterial calcification of infancy (mimicking severe coarctation of the aorta) with *ABCC6* gene variant — Case report and literature review

**DOI:** 10.3389/fcvm.2022.1032519

**Published:** 2022-12-20

**Authors:** Amalia Fãgãrãşan, Liliana Gozar, Simina-Elena Rusu Ghiragosian, Mircea Murariu, Marian Pop, Andrei Crauciuc, Diana Miclea, Carmen Corina Şuteu

**Affiliations:** ^1^Clinic of Pediatric Cardiology, Emergency Institute of Cardiovascular Diseases and Transplantation, Târgu Mureş, Romania; ^2^Department of Pediatrics III, George Emil Palade University of Medicine, Pharmacy, Science, and Technology of Târgu Mureş, Târgu Mureş, Romania; ^3^ME1 Department, George Emil Palade University of Medicine, Pharmacy, Science, and Technology of Târgu Mureş, Târgu Mureş, Romania; ^4^Department of Radiology, Emergency Institute for Cardiovascular Diseases and Heart Transplant, Târgu Mureş, Romania; ^5^Department of Medical Genetics, “Iuliu Hatieganu” University of Medicine and Pharmacy, Cluj-Napoca, Romania

**Keywords:** GACI, infantile heart failure, systemic hypertension, infant, *ABCC6* gene

## Abstract

**Introduction:**

Generalized arterial calcification of infancy (GACI) is a rare cause of infantile heart failure and systemic hypertension with a poor prognosis, characterized by extensive calcification and proliferation of the intimal layer of large and medium sized arteries.

**Case report:**

We present the first case report of successful surgical treatment of severe aortic arch obstruction by calcified plaques mimicking severe coarctation of the aorta and the outcome (of bisphosphonate therapy) in a newborn with GACI. Furthermore, we report the identification of a variant in ATP Binding Cassette Subfamily C, Member 6 (ABCC6) gene, possibly associated with severe early-onset manifestations of GACI.

**Conclusion:**

This case report highlights the importance of considering GACI in an infant with heart failure, systemic hypertension, and evidence of increased echogenicity of the arterial vessels. We noted the favorable outcome in improving the aortic calcification in our patient after surgical treatment and bisphosphonates therapy. Early diagnosis and treatment improve the long-term prognosis. A better understanding of this rare genetic disease could lead to new therapeutic strategies.

## Introduction

We present a case of a newborn with early-onset manifestations of generalized arterial calcification of infancy (GACI-2) mimicking severe coarctation of the aorta (CoA), and the clinical evolution of the patient in the first 18-months of life. To our knowledge, this is the first case report of successful surgical treatment of the aortic arch in a child with GACI. Furthermore, this case contributes to enrich the literature with data on a homozygote variant in *ABCC6* gene as-sociated with severe GACI phenotype.

## Case report

### Introduction

Generalized arterial calcification of infancy (GACI) is an autosomal recessive condition characterized by extensive calcification and intimal proliferation of the large and medium arteries, including the aorta, coronary arteries, and renal arteries, leading to vascular stenosis (1, 2). The most frequent complications include severe systemic hypertension, heart failure, myocardial infarction, respiratory distress, and sudden death. The disease was first described in 1901 by Bryant and White (3). This genetic disorder is caused by mutation in ectonucleotide pyrophosphatase/phosphodiesterase 1 (*ENPP1*) gene (GACI-1) and by mutation in ATP-binding cassette, subfamily C, member 6 (*ABCC6*) (GACI-2), leading to decreased levels of inorganic pyrophosphate (PPi). Inorganic pyrophosphate prevents deposition of calcium hydroxyapatite crystal in the vessel wall (4).

Generalized arterial calcification of infancy is a rare genetic disorder with a poor prognosis. Data about this condition is mainly based on case reports. To date, approximately 300 cases of GACI have been re-ported, and an estimated frequency of 1/566.000 has been suggested (5, 6). Mortality rate is high in infancy, 55% of children with GACI die within the first 6 months of life due to rapid progression of arterial stenoses and heart failure (1).

We present a case of a newborn with early-onset manifestations of GACI-2 mimicking severe CoA, and the clinical evolution of the patient in the first 18-months of life. CoAo is a congenital narrowing of the proximal descending aorta located mostly after the emergence of the left subclavian artery, being considered as the fifth most common congenital heart disease. Studies show the rate of recoarctation varies from 3 to 15% (7, 8). To our knowledge, this is the first case report of successful surgical treatment of the aortic arch in a child with GACI. Furthermore, this case contributes to enrich the literature with data on a homozygote variant in *ABCC6* gene as-sociated with severe GACI phenotype.

### Case report – The history of this case without 80-81, 177

We report the case of an 18-day-old male newborn referred from the neonatal intensive care unit of a peripheral hospital to our tertiary center for management of a CoA with ductal dependent systemic blood flow. It was related negative medical family history, the absence of consanguineous relations and no abnormality on the prenatal ultrasound. The boy was born at 38 weeks of gestation *via* cesarean section (for placental pathology), with a birth weight of 3,380g, and APGAR score of 10 at 1 min.

In the third day of life, the newborn developed heart failure and severe hypertension and the echocardiography revealed severe CoA. The diagnosis was confirmed by computed tomography (CT). Intravenous infusions of prostaglandin E1 (PGE1) was started in dose of 0.05 microgram/kg/min for keeping the ductus arteriosus (PDA) open. In this condition was admitted in our center.

### Presenting symptoms

The physical examination showed severe respiratory distress, a respiratory rate of 70 breaths per minute, cyanosis, with oxygen saturation on pulse oximetry (SaO2) of 85% in room air, tachycardia of 180 beats per minute, a grade III/IV systolic murmur with interscapulovertebral irradiation, weak pulse, a capillary refill time of 5 s, hepatomegaly, high systolic blood pressure (BP), ranging from 93 to 115 mmHg, and diastolic blood pressures between 40 and 50 mmHg (≥95th percentile for age); there was a significant difference in blood pressure between the upper and lower extremity (right upper limb BP 113/51 mmHg, left lower limb BP 64/31 mmHg).

**FIGURE 1:**
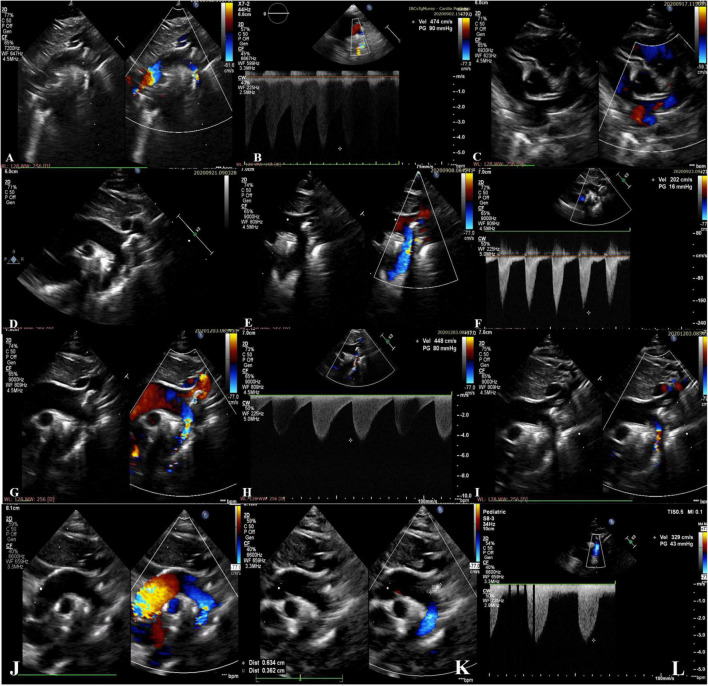
**(A–C)** Preoperative echocardiographic images from the neonatal period showing hyperechogenicity and severe narrowing of the aortic arch with a 2.5 mm diameter of the descending aorta lumen (gradient of 90mmHg) and normal coronary arteries; **(D–F)** postoperative echocardiographic images from the neonatal period showing the brightness and the abnormal echogenicity of the walls of the aortic arch and descending aorta and calcified residual masses at the level of the aortic arch extended to the brachiocephalic trunk, left common carotid artery, left subclavian artery, with laminar color Doppler flow in the descending aorta. Diameter of descending aorta lumen of 5.5 mm; **(G–I)** echocardiographic images at 3 months of age showing re-coarctation of the aorta with a significant gradient and the persistence of the aortic arch calcifications; **(J–L)** echocardiographic images at 18-months of age showing the regression of the aortic arch calcifications without obstruction, and mild left ventricular hypertrophy.

Transthoracic echocardiography showed left ventricular hypertrophy with impaired systolic function (LV ejection fraction of 40%), severe pulmonary hypertension, hyperechogenicity and severe narrowing of the aortic arch and descending aorta, with a significant gradient of 90 mmHg (Figures 1A,B). The coronary arteries were normal (Figure 1C). Ductus arteriosus was patent, but restricted, requiring an increase in PGE1 dose.

### Therapeutic approach, postprocedural evolution

**FIGURE 2:**
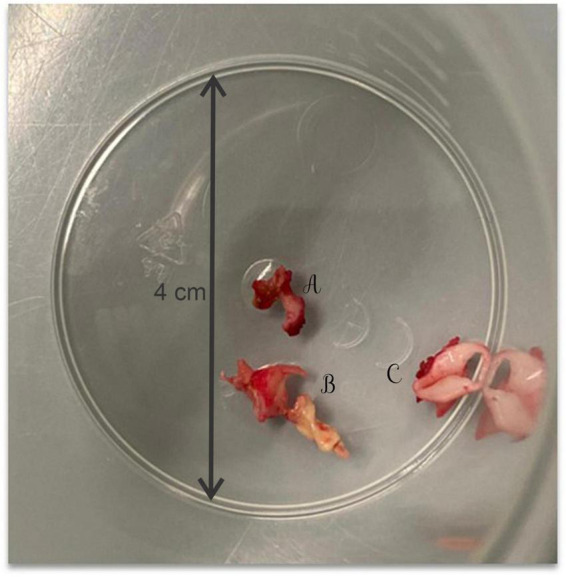
The calcified mass extracted intraoperatively from the left subclavian artery **(A)**, the aortic arch **(B)**, and the left common carotid artery **(C)**.

Due to hemodynamic instability, it was decided to perform the surgical repair of the CoA *via* lateral thoracotomy. Intraoperatively, after aortotomy, a calcified mass extended to the aortic arch, the left subclavian artery (LSA) and the left common carotid artery (LCCA) was detected and partially removed, and an end-to-end anastomosis was performed (Figure 2).

**FIGURE 3:**
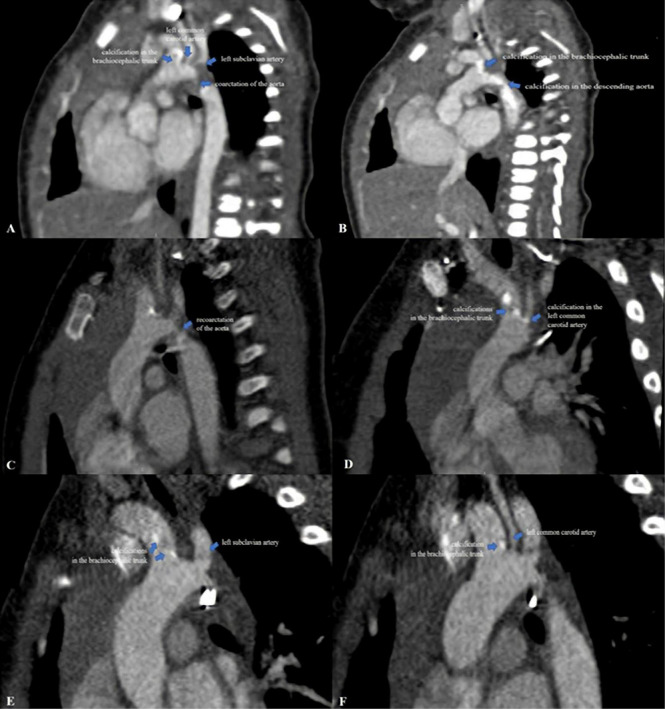
**(A,B)** Computed tomography images from the neonatal period showing extensive ectopic calcifications in the aortic arch, brachiocephalic trunk, left common carotid artery, left subclavian artery and descending aorta with severe narrowing of the aorta mimicking coarctation of the aorta. Note the brightness of the walls of the aortic arch and descending aorta; **(C,D)** computed tomography images at 3-months of age showing re-coarctation of the aorta and residual calcifications in the brachiocephalic trunk and left common carotid artery without obstruction; **(E,F)** computed tomography images at 18-months of age showing the regression of the aortic arch calcifications without stenosis.

Postoperative hemodynamic evolution was favorable (LV ejection fraction of 65%), the inotropic therapy being stopped in the first postoperative day. Despite the progressive clinical improvement, without a difference in BP between the upper and lower extremity (right upper limb BP 90/46 mmHg, left lower limb BP 80/40 mmHg) his systemic BP remained high (110–115 mmHg), and beta blocker therapy (Propranolol 1 mg/kg/day) was started. Postoperative echocardiography showed the brightness and the abnormal echogenicity of the walls of the aortic arch and descending aorta and calcified residual masses at the level of the aortic arch, with a 9 mm extension to the brachiocephalic trunk (BT), LCCA, LSA, with laminar color Doppler flow in the aorta (Figures 1D–F). The presence of ectopic calcification in the aorta was not appreciated preoperatively. Both preoperative ultrasound images and CT acquisitions were retrospectively reviewed (Figure 3).

Extended areas of increased echogenicity (compatible with calcifications) throughout the aortic arch extending into BT, LCCA, LSA, and descending aorta with severe narrowing of the aorta were noted (Figures 3A,B). To further elucidate the extent of the calcifications, whole-body CT scan was performed postoperatively, which detected diffuse areas of residual calcifications in the aortic arch, BT and LCCA without obstruction. No arterial calcifications were detected on the CT scan of the abdomen and head. Moreover, a histological exam of the calcified mass extracted intraoperatively was performed. This investigation highlighted a thickening of the vascular structure with fibrointimal proliferation and areas of nodular calcification and no histological appearance of thrombus. Parathyroid hormone, vitamin D3, calcium and phosphorus serum levels were normal (Table 1).

**TABLE 1:** The values of calcium and phosphate before and after bisphosphonate treatment.

Treatment	Calcium (mmol/L)	Phosphate (mmol/L)
Before treatment	2.72	2.17
After treatment	2.72	1.9

### Genetic investigation

The combination of vascular hyperechogenicity, severe aortic calcification with critical obstruction of the aortic arch mimicking CoA, and arterial hypertension determined us to consider GACI as a possible underlying etiology. The whole exome sequencing using Illumina HiSeq PE150 platform and SureSelect Human All Exon V6 kit was performed. The analysis of the results revealed the homozygous variant genotype for c.*1896C* > *A* (p. His632Gln) in exon 15 of the *ABCC6* gene. Also, the sequencing data analysis did not reveal any mutation in *ENPP1* gene. Furthermore, the molecular screening for thrombophilia revealed: methylene-tetrahydro-folate-reductase mutations (*MTHFR*)-heterozygous genotype *c.1298A* > *C*, and plasminogen activator inhibitor 1 (*PAI-1*)- heterozygous 4G/5G polymorphism.

### Pharmacotherapy and follow-up

Treatment with bisphosphonate was started. Zoledronic acid was administrated intravenously 0.025 mg/kg once every 3 months.

At the age of 3-months, his clinical course was complicated by aortic re-coarctation. The clinical exam revealed a grade III/IV systolic murmur with interscapulovertebral irradiation, bilateral absence of femoral pulse and a 20 mmHg BP difference between the upper and lower extremity (right upper limb BP 127/72 mmHg, left lower limb BP 105/60 mmHg). Echocardiography revealed a significant narrowing of the aortic isthmus (Figures 1G–I). CT scan confirmed the aortic re-coarctation and showed improvement of his residual aortic arch calcifications (Figures 3C,D). The infant underwent a prosthetic patch aortoplasty to enlarge the site of re-coarctation.

During the follow-up period, continuous monitoring with serial echocardiography was performed, emphasizing the reduction in size of aortic calcifications, especially at the level of BT. The child was re-admitted every 3 months for regular Zoledronic acid infusions. Antihypertensive medication was continued with regular blood pressure monitoring.

At the age of 18-months, the infant had normal growth and development; the BP was well controlled with Propranolol, without BP difference between the upper and lower extremity (right upper limb BP 100/61 mmHg, left lower limb BP 94/55 mmHg). Echocardiography showed that the vascular calcifications of the aortic arch and its branches were still present but were not progressing (Figures 1J–L), however, CT scan confirmed the regression of the aortic arch calcifications without stenosis (Figures 3E,F). There was no evidence of complications of his early calcification or of the bisphosphonate therapy.

### Results and case interpretation

The newborn we report was not initially clinically suspected of having GACI, considering that the preoperative clinical manifestations overlapped with the clinical course of severe CoA with restrictive PDA and systemic hypertension. Increased echogenicity of the aortic arch and its branches was not noted preoperatively by echocardiography and CT scan. Postoperatively, after the partial removal of the obstructive calcified plaque from the aortic arch, the increased echogenicity of the aortic arch and the residual calcifications could be much better appreciated by echocardiography. The combination of clinical features and extensive calcification of the aortic arch and its branches revealed on imaging and intraoperatively, together with histopathological findings and genetic testing confirmed the diagnosis of GACI-2 with severe early-onset manifestations. Although the main manifestations of GACI are widespread calcification of the aorta and its branches, including the aortic arch, the significant calcified plaques deposited in the aortic arch obstructing systemic flow and mimicking CoA presented in our case have not been previously described. Genetic testing of our patient revealed a variant in *ABCC6* gene reported in ClinVar database as a benign variant (9). This single nucleotide variant (SNV) is located in functional MRP6_HUMAN domain and, in a previous study, Le Saux et al. revealed that the missense mutations identified were considered pathogenic (10). The frequency of this SNV in European population is 0.4907, according to gnomAD database (11). This SNV identified determine, in 632 protein sequence, the substitution of glutamine with histidine, and according to UniProt database, this amino acid substitution is associated with pseudoxanthoma elasticum (PXE) (12). Furthermore, another study identified this SNV as a neutral (non-disease-causing) variant in patients with PXE (9). The analysis of the pathogenicity scores for *c.1896C* > *A* in prediction platforms established a benign predictor effect according to EIGEN, EIGEN PC, a tolerated prediction according to DEOGEN2, LIST-S2, SIFT, and a damaging prediction according to FATHMM. Therefore, the variant identified in our case is classified as a variant of uncertain significance (VUS) according to the American Collage of Medical Genetics Classification: PS1, PM1, PP2, PP5, BA1, BP4, BP6. Using applied bioinformatic tools it was performed *in silico* analysis based on algorithms which analyze the DNA and protein sequence substitution (Poly-Phen2, PhD-SNP, SNPs&Go). The functional effect of the identified SNV in our case was predicted to be benign. To our knowledge this variant has not previously been reported in association with GACI.

In our case, both the surgical treatment of the cardiovascular complication and the medical therapy with bisphosphonates had favorable outcomes; the patient successfully survived infancy without heart failure and with systemic hypertension under effective control. Zoledronic acid, a potent nitrogen(N)-containing-bisphosphonate, was administrated intravenously, 0.025 mg/kg once every 3 months, in accordance with the recommendations for use of bisphosphonates in pediatrics (13). At the time of this report, the patient is 18-months old, still under treatment with intravenously Zoledronic acid; CT scan and ultrasound showed a significant regression of aortic arch calcifications (0.5 mm from 9 mm at the level of BT) without stenosis. No side effects of long-term administration of bisphosphonates were detected.

Informed consent was obtained from the parents of the patient, and this case report was approved by the Ethics Committees of the Institute of Cardiovascular Diseases and Transplantation (permission number: 7718/14.10.2020).

## Discussion

Ectopic mineralization is defined by the aberrant deposition of calcium-phosphate complexes in tissues, and particularly in blood vessels (14). Vascular calcification is a major cause of morbidity and mortality (15). The pathophysiology of the ectopic mineralization is poorly understood. In recent decades, improvements have been made in understanding the pathophysiological mechanisms, revealing that vascular calcification can be the consequence of both a high-calcium (Ca) and high-phosphorous (P) milieu and very well-organized biological processes, including an imbalance between osteochondrogenic signaling and anti-calcific events (15). Failure of anti-calcification processes due to loss or deficiency of mineralization inhibitors leads to vascular calcification (15). Inorganic pyrophosphate (PPi) is known as an endogenous inhibitor of biomineralization. Extracellular pyrophosphate is synthesized from extracellular ATP and has the role of preventing the formation and deposition of hydroxyapatite crystals. *ENPP1* and *ABCC6* are plasma membrane-associated proteins involved in PPi synthesis (4). *ENPP1* is the principal enzyme that generates PPi from ATP hydrolysis; *ABCC6* works upstream of *ENPP1* by mediating release of ATP (16). Mutations in the genes encoding these enzymes reduce the plasma concentration of PPi, resulting in reduced PPi (antimineralization factor)/Pi (inorganic phosphate: promineralization factor), which stimulates ectopic mineralization of the arterial vessels and other tissues (15). In children, there are two major prototypes of such disorders that associate spontaneous pathological arterial calcifications: PXE, a late-onset, slowly progressing disease with multisystemic clinical manifestations, and GACI, a more severe disease characterized by early-onset mineralization of the cardiovascular system.

Generalized arterial calcification of infancy is a rare autosomal recessive disorder characterized by congenital calcification of large and medium sized arteries. The calcification of arterial structures is initiated in the internal elastic lamina and extends into the intima and media, being accompanied by fibrous thickening of the intima, which causes luminal narrowing (17). There are two forms of GACI. In most cases (67%), causal mutations have been identified in the genes *ENPP1*(GACI-1) (1). There are many studies reporting the mutations in ENPP1 in patients with GACI (2, 4, 17–20). In 9% of cases, GACI results from biallelic inactivating variants in *ABCC6* (GACI-2) (1). Mutations in *ABCC6* typically cause PXE. Nitschke et al. studying 92 patients with severe, early-onset arterial calcification, identified biallelic pathogenic mutations in *ENPP1* in 62 patients and *ABCC6* mutations in 14 patients. Biallelic mutations in *ABCC6* were found in 8 cases of typical GACI that presented widespread calcifications of the aorta and coronary arteries; additionally, in six patients with critical GACI, monoallelic *ABCC6* mutations were detected. Thirteen different mutations in *ABCC6* were identified. Furthermore, it has been suggested that there is a genotype-phenotype overlap between GACI and PXE (21). Ferreira et al. reported the results of clinical, laboratory and molecular evaluations of 20 subjects with GACI who survived infancy; they identified 16 biallelic *ENPP1* variants and 6 *ABCC6* variants; the patients with *ABCC6* variants did not differ in the frequency or location of ectopic calcification compared with those having *ENPP1* variants (1). Further evidence of genetic heterogeneity in the pathogenesis of GACI is based on identification of affected individuals who lack variants in either *ENPP1* or *ABCC6* (1, 21). While it is widely recognized that individuals with pathogenetic variants in *ENPP1* or *ABCC6* can manifest GACI, there is evidence that associateVUS with the GACI phenotype (22). Mahajan et al. reported two VUS located on chromosome *20q11.21* and *16p11.2* in a patient with GACI (22). In our case, the whole genome sequencing analysis revealed homozygous variant genotype for c.1896C > A of *ABCC6* gene. This SNV has been reported in *ABCC6* initially in 2001 as a neutral (non-disease-causing) variant in both heterozygous and homozygous states in a cohort of 122 patients with PXE (10). To our knowledge, this variant has not previously been reported in association with GACI phenotype.

Diagnosis of GACI is suspected based on clinical and imaging findings. A definitive diagnosis involves identification of variants in *ENPP1* or *ABCC6* and/or typical histopathological findings. The clinical spectrum of the disease varies widely. The most affected arteries are the aorta, coronary, pulmonary, cerebral, mesenteric, and renal arteries. Early-onset disease (48% of cases) presents variably with fetal distress, severe heart failure, respiratory distress, pulmonary hypertension, and systemic hypertension and refers to cases that occur in the fetal period or within 1 month of birth (6, 23). Presentation of late-onset disease (52% of cases) refers to cases where the onset is 1 month after birth and the clinical manifestations include respiratory failure, congestive heart failure, vomiting, feeding difficulties, irritability, failure to thrive, fever, hypertension, and edema (6, 23). Nearly half of all children with GACI are diagnosed during infancy (4, 24). Prenatal diagnosis is extremely rare and should be suspected when there is evidence of polyhydramnios or a history of early neonatal death or when increased echogenicity is observed along large vessels by fetal ultrasound (4, 25). A small number of prenatally diagnosed cases of GACI have been reported in the literature (26, 27). In our case, antenatal ultrasonography did not reveal any abnormalities.

Generalized arterial calcification of infancy should be considered in infants with signs of congestive heart failure, myocardial ischemia, severe systemic hypertension, respiratory failure (4, 28). The diagnosis involves high index of suspicion. Limited awareness of this disease and low suspicion by physicians make early diagnosis rare. Cases clinically suspicious for GACI can be confirmed with imaging studies, arterial biopsy, and genetic analysis (29). Imaging investigations are extremely useful in the clinical diagnosis of GACI by identifying increased echogenicity of arterial walls and vascular narrowing. While echocardiography is useful, it may not provide a definitive diagnosis and additional imaging techniques such as whole-body CT scan combined with CT angiography are usually necessary to assess the extent of calcifications and stenoses in the arteries. Several clinical reports have shown extensive calcifications and narrowing of the aortic arch and descending aorta, but no case developed severe obstruction (20, 22, 23). The severe phenotype of GACI mimicking CoA presented in our case have not been previously described.

The prognosis of GACI patients is poor, the greatest number of deaths occurring within the first 6 months; most infants die from severe, rapidly progressive heart failure or myocardial infarction. Only a few cases of survivors into later childhood or adulthood are reported (30–32). O’Brien et al. evaluated the lifelong impact of *ENPP1* deficiency and the early onset form of *ABCC6* deficiency from a patient or caregiver perspective and concluded that these morbidities are debilitating diseases with lifelong morbidity, including pain and impaired mobility, and those who are affected experience impairment of quality of life and psychosocial issues throughout life (33).

Early appropriate diagnosis would allow treatment and is associated with a higher survival rate. There are no guidelines for GACI treatment. In recent years, basic science has elucidated the molecular pathway and mechanisms involved in PXE, which has led to rapid advances in the development of many potential therapeutic options for PXE and GACI (34). The therapeutic solutions target various steps in the *ABCC6* pathway with the goal of either slowing or reversing the progression of the disease. Two main categories of therapeutic solutions have been proposed, most of them have been tested in pre-clinical animal models and a few early clinical trials. The first therapeutic strategy targets correction, replacement, or inhibition of dysfunctional genes/proteins [*ABCC6*, ectonucleotides (NPP1) and tissue non-specific alkaline phosphatase (TNAP)] (34). Nitschke et al. concluded that ENPP1 enzyme replacement by subcutaneous administration of the rhENPP1-Fc protein [recombinant human (rh)ENPP1-Fc protein] may influence vascular smooth muscle cells proliferation and may serve as an approach for effective prevention and treatment of arterial stenosis in GACI (35). The second therapeutic strategy targets direct inhibition of calcification *via* supplementation by various compounds: magnesium (inhibits the formation of apatite), vitamin K (correct for insufficient carboxylation of matrix gla protein), bisphosphonates (inhibit enzymes that utilize pyrophosphate), pyrophosphate (potent inhibitor of calcification, *ABCC6* modulates PPi production), phytic acid (inhibitor of calcification), sodium thiosulfate (approved for calciphylaxis) (34).

Bisphosphonates are PPi analogs and have been shown to decrease phosphate levels, increase parathormone levels and cause stabile levels of calcium (36). Bisphosphonates have been used clinically for decades in treatment of osteoporosis, Paget’s disease of bone, and osteogenesis imperfecta (34). The results of bisphosphonate therapy in GACI are variable, spontaneous resolution of calcifications on CT scan or echocardiography has occasionally been reported, but the long-term prognosis in survivors is not described (5, 37). In their retrospective study, Rutsch et al. reported that bisphosphonate therapy was associated with survival in 11 (65%) of 17 treated patients whereas 69% of the patients not treated with bisphosphonates died (38). Ramjan et al. reported complete resolution of arterial calcification with the long-term usage of bisphosphonates (39). Weingarten et al. recently reported the favorable therapeutic response in a case of GACI using an innovative therapeutic plan including bisphosphonate (Pamidronate), Acetazolamide (increase urinary excretion of phosphate) and Similac PM 60/40 (a low calcium and phosphate formula) (37). Yunfeng et al. reported the case of a preterm infant with GACI and concluded that the treatment with oral phosphonates is expected to improve the long-term prognosis (23).

There are two categories of bisphosphonates based on the presence of a non-nitrogenous or nitrogenous side chain. Etidronate is a non-nitrogen(N)-containing-bisphosphonate whose antiresorptive potency is at the lower end of the scale, while Pamidronate, Alendronate, Risedronate and Zoledronic acid are more potent N-containing-bisphosphonates. Furthermore, N-containing-bisphosphonates usually increase bone density, demonstrating a better antifracture efficacy (34). Etidronate has been used to treat GACI patients and early treatments improved GACI outcomes. Prolonged Etidronate use in GACI patients has been associated with severe skeletal toxicity, radiographic findings resembling osteoporosis or hypophosphatemia (40). It remains unclear whether bisphosphonates, Etidronate in particular, are associated with improved survival (41).

Zoledronic acid is being developed as an intravenous therapy and has the highest affinity for hydroxyapatite and the longest duration of action. Mixed results associated with Zoledronic acid treatment have been reported. Synetos et al. in an experimental model of aortic stenosis, concluded that the inhibition of aortic valve calcification by local delivery of Zoledronic acid was feasible and effective, without evident short-term complications (42). Cai et al. demonstrated that once-yearly Zoledronic acid did not affect progression of abdominal aortic calcification over 3 years in postmenstrual women with osteoporosis (43). The anti-calcification properties of Zoledronic acid for PXE have been reported in an *in vitro* series of experiments with primary fibroblasts (44). Intravenous Zoledronic acid or Pamidronate are the treatment of choice in patients with moderate-to-severe osteogenesis imperfecta (45). In our case, we opted for the treatment with Zoledronic acid considering the severe phenotype of the disease as well as the superior pharmacokinetics of this N-bisphosphonate, which is essential for optimal clinical outcomes and minimalization of the risk of adverse effects.

Deshpande et al. published the first report of successful mechanical support in a patient with GACI, additionally, over time with bisphosphonate therapy, there was a remarkable recovery of cardiac function (46). Giovannoni et al. reported an 18-month-old child with GACI and end-stage myocardial ischemia who underwent successful heart transplantation (47). Although Samyn et al. reported the first case of successful cardiac surgery in a child with severe pulmonary valve stenosis and GACI (48, 49), until now no one has reported surgical treatment for associated severe obstruction of the aortic arch in a newborn with GACI. In our case, both the surgical treatment of the cardiovascular complication and the medical therapy with Zoledronic acid had favorable outcomes. The patient successfully survived infancy with systemic hypertension under effective control, without bone or joint modifications, no signs of heart failure and significant regression of aortic arch calcifications on imaging.

## Conclusion

Our case highlights the importance of considering GACI in an infant with heart failure, systemic hypertension, and evidence of increased echogenicity of the arterial vessels on imaging. Early diagnosis and treatment improve the long-term prognosis. We noted the favorable outcome in improving the aortic calcification in our patient after surgical treatment of the severe aortic arch obstruction by calcified plaques and bisphosphonates therapy. Furthermore, we report the identification of a variant in *ABCC6* gene, possibly associated with severe early-onset manifestations of GACI mimicking severe CoA. A better understanding of this rare genetic disease and further evidence of genetic heterogeneity both by identifying affected individuals who lack variants in either ENPP1 or ABCC6 and elucidating possible genes involved in this condition could lead to new therapeutic strategies.

## Data availability statement

The original contributions presented in the study are publicly available. This data can be found at the European Nucleotide Archive, accession number: PRJEB57858.

## Author contributions

CŞ: writing – original draft preparation. CŞ, LG, and AC: writing – review and editing. S-EG, MM, and MP: visualization. AF: supervision. DM: performing and interpreting genetic testing. All authors read and agreed to the published version of the manuscript.
